# BMI and Cardiometabolic Traits in Japanese: A Mendelian Randomization Study

**DOI:** 10.2188/jea.JE20220154

**Published:** 2024-02-05

**Authors:** Mako Nagayoshi, Asahi Hishida, Tomonori Shimizu, Yasufumi Kato, Yoko Kubo, Rieko Okada, Takashi Tamura, Jun Otonari, Hiroaki Ikezaki, Megumi Hara, Yuichiro Nishida, Isao Oze, Yuriko N. Koyanagi, Yohko Nakamura, Miho Kusakabe, Rie Ibusuki, Keiichi Shibuya, Sadao Suzuki, Takeshi Nishiyama, Teruhide Koyama, Etsuko Ozaki, Kiyonori Kuriki, Naoyuki Takashima, Yasuyuki Nakamura, Sakurako Katsuura-Kamano, Kokichi Arisawa, Masahiro Nakatochi, Yukihide Momozawa, Kenji Takeuchi, Kenji Wakai

**Affiliations:** 1 Department of Preventive Medicine, Nagoya University Graduate School of Medicine, Nagoya, Japan; 2 Undergraduate Course, Nagoya University Graduate School of Medicine, Nagoya, Japan; 3 Department of Psychosomatic Medicine, Graduate School of Medical Sciences, Kyushu University, Fukuoka, Japan; 4 Department of Psychosomatic Medicine, International University of Health and Welfare Narita Hospital, Chiba, Japan; 5 Department of General Internal Medicine, Kyushu University Hospital, Fukuoka, Japan; 6 Department of Comprehensive General Internal Medicine, Kyushu University Faculty of Medical Sciences, Fukuoka, Japan; 7 Department of Preventive Medicine, Faculty of Medicine, Saga University, Saga, Japan; 8 Division of Cancer Epidemiology and Prevention, Aichi Cancer Center Research Institute, Nagoya, Japan; 9 Division of Cancer Information and Control, Aichi Cancer Center Research Institute, Nagoya, Japan; 10 Cancer Prevention Center, Chiba Cancer Center Research Institute, Chiba, Japan; 11 Department of International Island and Community Medicine, Kagoshima University Graduate School of Medical and Dental Sciences, Kagoshima, Japan; 12 Department of Emergency, Kagoshima Prefectural Oshima Hospital, Kagoshima, Japan; 13 Department of Public Health, Nagoya City University Graduate School of Medical Sciences, Nagoya, Japan; 14 Department of Epidemiology for Community Health and Medicine, Kyoto Prefectural University of Medicine, Kyoto, Japan; 15 Laboratory of Public Health, Division of Nutritional Sciences, School of Food and Nutritional Sciences, University of Shizuoka, Shizuoka, Japan; 16 Department of Public Health, Faculty of Medicine, Kindai University, Osaka, Japan; 17 Department of Public Health, Shiga University of Medical Science, Otsu, Japan; 18 Yamashina Racto Clinic and Medical Examination Center, Kyoto, Japan; 19 Department of Preventive Medicine, Institute of Biomedical Sciences, Tokushima University Graduate School, Tokushima, Japan; 20 Public Health Informatics Unit, Department of Integrated Health Sciences, Nagoya University Graduate School of Medicine, Nagoya, Japan; 21 Laboratory for Genotyping Development, Center for Integrative Medical Sciences, Kanagawa, Japan; 22 Department of International and Community Oral Health, Tohoku University Graduate School of Dentistry, Sendai, Japan

**Keywords:** body mass index, diabetes mellitus, cardiometabolic risk factors, Mendelian randomization analysis, East Asian people

## Abstract

**Background:**

Although many observational studies have demonstrated significant relationships between obesity and cardiometabolic traits, the causality of these relationships in East Asians remains to be elucidated.

**Methods:**

We conducted individual-level Mendelian randomization (MR) analyses targeting 14,083 participants in the Japan Multi-Institutional Collaborative Cohort Study and two-sample MR analyses using summary statistics based on genome-wide association study data from 173,430 Japanese. Using 83 body mass index (BMI)-related loci, genetic risk scores (GRS) for BMI were calculated, and the effects of BMI on cardiometabolic traits were examined for individual-level MR analyses using the two-stage least squares estimator method. The β-coefficients and standard errors for the per-allele association of each single-nucleotide polymorphism as well as all outcomes, or odds ratios with 95% confidence intervals were calculated in the two-sample MR analyses.

**Results:**

In individual-level MR analyses, the GRS of BMI was not significantly associated with any cardiometabolic traits. In two-sample MR analyses, higher BMI was associated with increased risks of higher blood pressure, triglycerides, and uric acid, as well as lower high-density-lipoprotein cholesterol and eGFR. The associations of BMI with type 2 diabetes in two-sample MR analyses were inconsistent using different methods, including the directions.

**Conclusion:**

The results of this study suggest that, even among the Japanese, an East Asian population with low levels of obesity, higher BMI could be causally associated with the development of a variety of cardiometabolic traits. Causality in those associations should be clarified in future studies with larger populations, especially those of BMI with type 2 diabetes.

## INTRODUCTION

Cardiovascular diseases (CVDs), both ischemic heart disease and stroke, are leading causes of death worldwide. Ischemic heart disease was responsible for 16% of the world’s total deaths in 2019, showing the largest increase from 2000 (more than 2 million) to 2019 (8.9 million deaths), while stroke was responsible for approximately 11% of total deaths globally.^1^

Observational studies have shown that obesity and higher BMI is the most prominent risk factor for CVDs,^2^ being associated with the development of atherosclerosis^3^ via obesity-related dyslipidemia,^4^ type 2 diabetes,^4^^–^^6^ hypertension,^4^^,^^7^ and kidney dysfunction^8^ through various mechanisms,^9^^–^^11^ including inflammation.^12^^,^^13^ The level of inflammation in obese people tends to be higher than that in the non-obese.^14^^–^^16^ Atherosclerosis is also a state of chronic systemic inflammation. Obesity accelerates inflammation because adipose tissue produces proinflammatory adipokines (eg, TNF-alpha, IL-6, monocyte chemoattractant protein-1, resistin, and leptin),^14^^,^^15^^,^^17^ which are directly involved in atherosclerosis.^16^ In line with the rapid increase of obesity worldwide in the past decades,^18^ cardiometabolic diseases due to obesity are an ongoing major public health burden. Against the background of the novel coronavirus disease 2019 pandemic, CVDs due to obesity could become a more critical issue because of decreased physical activity and the adoption of increasingly sedentary lifestyles.

Although many observational studies have demonstrated the significant relationships between obesity or higher BMI and cardiometabolic diseases and their risk factors,^4^^–^^6^ the causal relationships have not been fully elucidated because the mechanisms involved are complex.^19^ Not only behavioral factors (eg, diet, sleep patterns, and sedentary lifestyle) and biological factors (eg, hormonal, nutritional, and metabolic factors), but also genetic factors (eg, fat mass and obesity-associated gene [FTO]),^20^ environmental factors (eg, socioeconomic status, culture, body norms,^21^^,^^22^ walkability of the neighborhood,^23^ and urbanity^24^) and psychological factors (eg, mental stress) are related to the pathogenesis of both obesity and cardiometabolic diseases. It is also difficult to take into account unconscious bias, reverse causation, and interactions between individual (including genetic) and environmental factors in both conditions.

Mendelian randomization (MR) is a novel epidemiological approach that uses genetic variants as instrumental variables (IVs). MR makes a causal inference in observational data without the influence of confounders, through the random assortment of genetic variants during meiosis.^25^ Although recent studies have revealed the causal relationships between obesity and cardiometabolic diseases in Europeans by MR,^26^^,^^27^ limited evidence is currently available in East Asians, including Japanese. Considering the differences in genetic and environmental backgrounds, and in the prevalence of obesity (defined as BMI ≥30 kg/m^2^) between Japan (3.7%) and the United States (38.2%),^18^^,^^28^ it is important to clarify the causal relationships between BMI and cardiometabolic diseases in each of these populations. Because the largest Japanese genome-wide association study (GWAS) performed to date reported significant genetic correlations between BMI and some cardiometabolic traits, such as ischemic stroke, myocardial infarction, and type 2 diabetes,^29^ we thought that MR studies for these conditions may clarify the associated directions of causality among the Japanese. Accordingly, to investigate whether obesity defined by genetically determined BMI can affect the risk of CVD and related cardiometabolic traits in Japanese, we conducted the MR study in the Japanese population.

## METHODS

### Study data for individual-level MR

**Figure 1.**
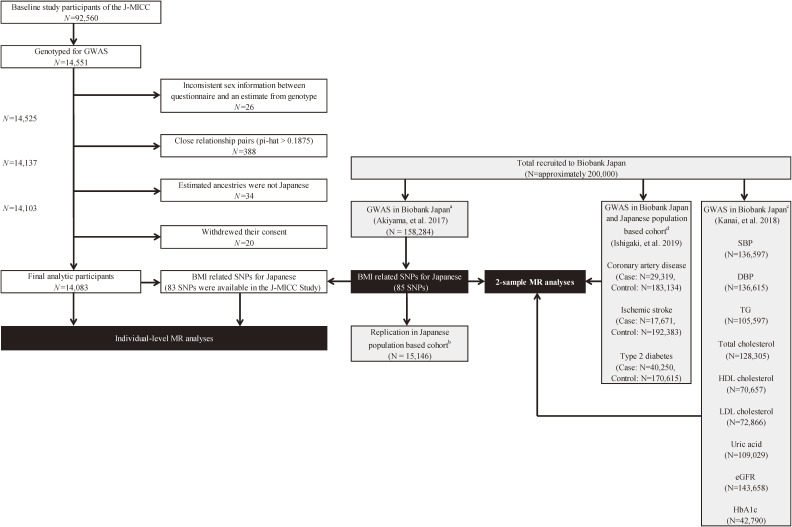
Study sample flow chart. BBJ, BioBank Japan Project; DBP, diastolic blood pressure; eGFR, estimated glomerular filtration rate; HbA1c, hemoglobin-A1c; HDL cholesterol, high-density-lipoprotein cholesterol; J-MICC Study, Japan Multi-institutional Collaborative Cohort Study; LDL cholesterol, low-density-lipoprotein cholesterol, SBP, systolic blood pressure; SD, standard deviation; TG, triglyceride. ^a^Detailed information is available in the previous report by Akiyama et al. ^b^Participants from the Japan Public Health Center-based Prospective Study (JPHC) and the Tohoku Medical Megabank (TMM) Project were included in the replication. ^c^Detailed information is available in the previous report by Kanai et al. ^d^Each case was from the BioBank Japan Project (BBJ), and each control was from among the rest of the participants of the BBJ and those of four Japanese cohorts (Tohoku Medical Megabank [TMM], Iwate Tohoku Medical Megabank [IMM], JPHC, and J-MICC Study). Detailed information is available in the previous report by Ishigaki et al.

For individual-level MR analyses in this study, we targeted participants in the Japan Multi-Institutional Collaborative Cohort (J-MICC) Study. This is one of the largest genome cohort studies performed in Japan, which recruited 92,560 participants nationwide from 14 study areas of Japan between 2004 and 2014. In this study, informed consent was obtained from the participants, followed by a self-administered questionnaire survey on lifestyle and medical history, along with blood sampling and anthropometric measurements. Blood samples were collected using a 7 mL vacuum tube for serum and a 7 mL EDTA-2Na-containing vacuum tube for plasma and buffy coat. Collected baseline data and blood samples were anonymized in each of the participating institutions and then sent to the central executive office at Nagoya University. Among all participants, 14,551 eligible participants who were selected for GWAS from 13 study areas throughout Japan were genotyped. The genotyping was conducted at the RIKEN Institute (Yokohama, Japan) using an Illumina OmniExpressExome Array (Illumina, San Diego, CA, USA) for 964,193 single-nucleotide polymorphisms (SNPs). We excluded 26 participants for whom information on their sex was inconsistent between the questionnaire and the genotype results. We detected 388 close relationship pairs (pi-hat >0.1875) that were detected using the identity-by-descent method of PLINK 1.9 software (https://www.cog-genomics.org/plink2) and excluded one sample of each pair. Principal component analysis^30^ with a 1000 Genomes reference panel (phase 3) (http://www.internationalgenome.org/category/phase-3/) detected 34 subjects whose estimated ancestries were outside of the Japanese population, so their samples were excluded. All of the remaining 14,103 samples met the sample-wise genotype call rate criterion (≥0.99). SNPs with a genotype call rate <0.98 and/or a Hardy–Weinberg equilibrium exact test *P* value <1 × 10^−6^, a low minor allele frequency (MAF) <0.01, or departure from the allele frequency computed from the 1000 Genomes Phase 3 EAS (East Asian) samples were excluded. Quality control filtering resulted in 14,103 individuals with 570,162 SNPs. From among these individuals, we excluded 20 participants who withdrew from the study, leaving 14,083 individuals for the final analyses (Figure 1). Genotype imputation was conducted using SHAPEIT ver. 2 (https://mathgen.stats.ox.ac.uk/genetics_software/shapeit/shapeit.html#home) and Minimac3 (http://genome.sph.umich.edu/wiki/Minimac3) software based on the 1000 Genomes Project cosmopolitan reference panel (phase 3).

The blood biochemistry data, such as serum triglycerides (TG), total cholesterol (TC), high-density-lipoprotein (HDL) cholesterol, low-density-lipoprotein (LDL) cholesterol, glycated hemoglobin (HbA1c), uric acid (UA), and creatinine levels, were collected from health check-ups conducted in each of the participating areas. LDL was estimated using the following formula by Friedwald et al^31^: [TC (mg/dL) − HDL (mg/dL)] − TG (mg/dL) × 0.2, if serum TG level was less than 400 mg/dL; otherwise, it was treated as missing. The estimated glomerular filtration rate (eGFR) was calculated using the following formula, which was specifically derived for the Japanese population: eGFR (mL/min/1.73 m^2^) = 194 × serum creatinine^−1.094^ × age^−0.287^ (if female, × 0.739).^32^ Blood pressure of each participant was measured using a standard mercury sphygmomanometer or an automated measurement monitor by trained medical staff, with each participant in a seated position. Coronary artery disease (CAD) and ischemic stroke (IS) were detected using self-reported medical histories. Type 2 diabetes was detected if participants had a self-reported diagnosis of diabetes, took blood glucose-lowering medications, and/or had HbA1c of 6.5% or higher at baseline.

The protocol of the J-MICC Study was approved by the ethics review committees of Nagoya University Graduate School of Medicine (Approval No. 939-14), Aichi Cancer Center, and all participating institutions. All research procedures were conducted in accordance with the Ethical Guidelines for Human Genome and Genetic Sequencing Research in Japan and the Declaration of Helsinki.

### Instruments for BMI as exposure and outcome phenotypes

We adopted the lead SNPs in 85 loci that were discovered to be strongly associated with BMI in a recent Japanese GWAS.^29^ As only 75 were available in the J-MICC GWAS among these 85 loci,^29^ we also used proxy SNPs for 8 out of 10 unavailable SNPs (2 SNPs [rs4308481 and rs180950758] are still unavailable), and provided for the individual-level MR analyses. The proxy variants were searched for using the LDlink (https://ldlink.nci.nih.gov/?tab=home), which is based on the 1000 Genomes JPT (Japanese in Tokyo) reference panel. Based on the 1000 Genomes JPT reference panel, the linkage disequilibrium (LD) between the proxy SNPs and the original variants were perfect other than one SNP—rs79823890 for rs75766425 (*D*′ = 0.95, *r*^2^ = 0.86). Using the BMI-related SNPs, we calculated a weighted genetic risk score (GRS) for BMI in the Japanese, where each allele dosage was weighted by the per-allele change in 1 unit increase of BMI. The weighted GRS was used as an IV for individual-level MR analyses. Detailed information of the summary statistics for the SNPs adopted as IVs is presented in eTable 1. We performed the MR analysis assuming that IVs is associated with the exposure of interest, without sharing no unmeasured cause with outcome, and affect outcome only through its potential effect on the exposure of interest.^33^ We checked the 85 SNPs adopted as IVs whether they also have strong association with other cardiometabolic traits in GWAS Catalog (https://www.ebi.ac.uk/gwas/). The number of total SNPs (number of SNPs for Asians or East Asians) fulfilled the GWAS significance (*P* < 5 × 10^−8^) in the association was 10 (7) SNPs for type 2 diabetes (rs633715, rs6947395, rs10795945, rs80234489, rs11642015, rs6567160, rs1379871 for Asians/East Asians), 3 (2) SNPs for SBP (rs7903146, rs11642015), 2 (1) SNPs for DBP (rs4409766), 3 (1) SNPs for TG (rs75766425), 2 (0) SNPs for HDL cholesterol and 1 (1) SNPs for HbA1c (rs35261542). In sensitivity analyses, we excluded those 12 SNPs for Asians in the MR analyses to exclude the pleiotropy effects.

### Study data for two-sample MR

We leveraged the summary statistics of the recently published GWAS meta-analysis of Japanese BMI,^29^ which is based on the GWAS data of BioBank Japan (BBJ) (*N* = 158,284), and those of the Japan Public Health Center-based Prospective Study (JPHC study) (*N* = 7,379) and Tohoku Medical Megabank (TMM) (*N* = 7,767). BBJ is the largest hospital-based case-control study to have been performed in Japan, which enrolled approximately 200,000 participants with 47 disease outcomes throughout the country. The JPHC study and TMM are the two major population-based genome cohorts in Japan. For the second sample, the GWAS summary statistics of cardiovascular diseases and various cardiometabolic traits phenotypes in the Japanese were referenced from the data repository of Jenger (http://jenger.riken.jp/), which is the database for BBJ GWAS (quantitative traits from Kanai et al^34^ and binary disease traits from Ishigaki et al^35^). For the GWAS summary statistics of cardiovascular diseases and type 2 diabetes, each case was from BBJ, and each control was from among the rest of the participants of BBJ and participants of four Japanese cohorts; including TMM, Iwate Tohoku Medical Megabank, JPHC, and J-MICC Study^35^ (Figure 1).

### Statistical analysis for individual-level MR analyses

Descriptive statistics were generated for BMI-related variables and covariates, including measured BMI, systolic blood pressure (SBP), diastolic blood pressure (DBP), TG, TC, HDL cholesterol, LDL cholesterol, UA, eGFR, and HbA1c, as well as proportion of type 2 diabetes, and CVDs (CAD and IS). All quantitative traits with a skewed distribution were natural-logarithmically transformed to approximate univariate normality (ie, TG and LDL). The associations of BMI with those traits were examined by individual-level MR analysis. A weighted GRS for BMI was obtained from the following calculations: at first, the number of alternative alleles coded as (0,1,2), second, regression coefficients (β) of BMI on the number of alternative alleles with adjustments for age and sex were calculated for all the SNPs, and then the sum of the number of alternative alleles weighted by β was calculated. Then, the ivreg2 command of Stata (Stata Corp, College Station, TX, USA) was used with the weighted GRS as an IV. In the first stage regression, we regressed BMI on weighted GRS, and in the second stage, we regressed quantitative cardiometabolic traits on estimated BMI with adjustments for age and sex. For these analyses, the two-stage least squares estimator method was used. In the case of binary outcomes (CAD, IS and type 2 diabetes), we examined two-stage regressions by Burgess et al.^36^ Briefly, we regress BMI on SNPs (IVs) and then conduct the logistic regression analysis of each cardiometabolic trait on the genetically predicted BMI obtained.

To ensure the association between exposure and outcomes, we examined phenotypical associations between BMI and cardiometabolic traits using measured data from the J-MICC study. To assess the possibility of the violation of MR assumption by IV-confounder associations, we tested the associations of weighted GRS for BMI with smoking (Brinkman Index), drinking (alcohol consumption: grams of alcohol/day) and daily physical activities (metabolic equivalents of task [METs]/day) as potential confounders.

### Statistical analysis for two-sample MR analyses

We obtained the β-coefficients and standard errors for the per-allele association of each SNP as well as all outcomes from the above-mentioned data sources. Data are presented as the mean, standard deviation (SD), *n* (%), or odds ratio (OR) with 95% confidence interval (CI). The associations of BMI with CVDs and cardiometabolic traits were examined based on the summary statistics of Jenger using the two-sample MR approach. Two methods of two-sample MR—the Inverse-Variance Weighted (IVW) method and the MR–Egger method—were conducted as sensitivity analyses to test whether the results of IVs were robust. First, we calculated Wald ratios for each IV by dividing the per-allele log-odds ratio or beta of that variant in the outcome data (cardiometabolic traits from Jenger [http://jenger.riken.jp/]) by the log-odds ratio or beta of the same variant in the exposure data (BMI from Japanese GWAS meta-analysis^29^).

MR–Egger regression was conducted to assess the horizontal pleiotropy of the IVs, where the regression line is not constrained to pass through the origin, but the slope represents pleiotropy-corrected causal estimates. If the regression intercept (α) significantly differed from zero (*P* < 0.05), we considered that there was horizontal pleiotropy or that the InSIDE (Instrument Strength Independent of Direct Effect) assumption was violated.^37^ Heterogeneity between IVs in the IVW method was estimated using Cochran’s Q statistic. The β-coefficients and their 95% CIs for the two-sample MR analyses (IVW and MR–Egger) were calculated using Mendelian Randomization package of R (version 3.6.3; R Foundation for Statistical Computing, Vienna, Austria).

To assess the robustness of IVW-MR results, we conducted additional sensitivity analyses using the weighted median MR and Mendelian Randomization Pleiotropy RESidual Sum and Outlier (MR-PRESSO).^38^ The MR-PRESSO approach was used to rule out the possibility of false-positives for the outcome cardiometabolic phenotypes that had significant associations with BMI in the IVW-MR analyses. Briefly, we calculated the outlier-corrected exposure β values after excluding outliers based on the outlier test until the MR-PRESSO global test indicated no significant influence of outliers (Global-*P* ≥ 1.0 × 10^−6^).^38^^,^^39^ The MR-PRESSO analyses were conducted using MR-PRESSO package of R (https://github.com/rondolab/MR-PRESSO).

The Multivariable Mendelian Randomization (MVMR) was conducted to evaluate horizontal pleiotropy of IVs for BMI (eg, existence of other cardiometabolic pathways) in the associations with CHD and IS. MVMR was conducted using MVMR package of R (https://github.com/WSpiller/MVMR). A two-tailed *P* value <0.05 was considered nominally significant. For adjustments of multiple comparisons, we conducted the Bonferroni’s adjustments by the number of phenotypes in each analysis. For example, statistical significance threshold after the Bonferroni’s adjustment was set at a two-tailed *P* value of <0.05/12 = 0.00417 for individual MR and two-sample MR.

## RESULTS

### Characteristics of the participants for individual-level MR analyses

Table 1 shows the characteristics of the participants in the J-MICC Study. The final analyzed sample that was genotyped included 14,083 participants (57.2% female; mean age: 54.7 years; and mean BMI: 23.0 kg/m^2^). Participants with CVDs constituted 4.9% of the total (403 CAD and 248 IS) (Table 1).

**Table 1.** Characteristics of study participants who were genotyped in the J-MICC Study

Variables		Men		Women
		*N* = 6,336		*N* = 7,747
	*N* data		*N* data	
Age, years, mean (SD)	6,336	55.3 (9.3)	7,747	54.3 (9.4)
Body mass index, kg/m^2^, mean (SD)	6,335	23.8 (3.2)	7,733	22.5 (3.4)

Cardiometabolic binary traits, *N* (%)				
Coronary artery disease^a^	5,987	239 (4.0)	7,301	164 (2.3)
Ischemic stroke^a^	5,836	145 (2.4)	7,283	103 (1.4)
Type 2 diabetes^b^	6,336	769 (12.1)	7,747	416 (5.4)

Cardiometabolic quantitative traits, mean (SD)				
SBP, mm Hg	5,063	131.9 (19.2)	6,190	125.3 (20.4)
DBP, mm Hg	5,063	81.6 (11.8)	6,189	75.5 (11.9)
TG, mg/dL	5,327	149.6 (113.8)	6,318	109.3 (73.5)
Total cholesterol, mg/dL	4,883	205.6 (32.9)	5,808	215.9 (35.5)
HDL cholesterol, mg/dL	5,328	57.2 (15.0)	6,319	67.4 (15.9)
LDL cholesterol, mg/dL	4,882	122.3 (32.7)	5,807	127.4 (32.6)
Uric acid, mg/dL	4,898	6.0 (1.3)	5,895	4.4 (1.0)
eGFR, mL/min/1.73 m^2^	5,233	76.0 (15.1)	6,247	79.1 (16.1)
HbA1c, % (NGSP)	3,975	5.6 (0.9)	4,659	5.5 (0.7)

DBP, diastolic blood pressure; eGFR, estimated glomerular filtration rate; HbA1c, hemoglobin-A1c; HDL cholesterol, high-density-lipoprotein cholesterol; J-MICC Study, Japan Multi-institutional Collaborative Cohort Study; LDL cholesterol, low-density-lipoprotein cholesterol, SBP, systolic blood pressure; SD, standard deviation; TG, triglyceride.

^a^Coronary artery disease and ischemic stroke were detected from self-reported medical history.

^b^Type 2 diabetes was detected if participants had a self-reported diagnosis of diabetes, took blood glucose-lowering medications, and/or had HbA1c of 6.5% or higher at baseline.

### Individual-level MR analyses

The 83 loci associated with BMI in the Japanese population are shown in eTable 1. When we used the IVs, a higher weighted GRS of BMI was significantly associated with higher BMI (β = 1.817; 95% CI, 1.572–2.06; *P* < 0.001). The weighted GRS of BMI was not significantly associated with any cardiometabolic traits after the Bonferroni’s adjustment (significance level: *P* < 0.05/12 = 0.00417) (Table 2). The phenotypic associations assessed using measured data from the J-MICC study showed that BMI was significantly associated with all cardiometabolic traits other than eGFR (eTable 2). The weighted GRS for BMI was not associated with potential confounders of smoking (Brinkman Index), drinking (alcohol consumption: grams of alcohol/day) and daily physical activities (METS/day) (eTable 3).

**Table 2.** Associations of the predicted BMI^a^ and cardiometabolic traits by individual-level MR in the J-MICC Study

Trait	*β*	95% CI	*P*
BMI, kg/m^2 b^	1.817	(1.572–2.063)	<0.001
Coronary artery disease^c,d^	0.009	(−0.249 to 0.268)	0.632
Ischemic stroke^c,d^	0.092	(−0.226 to 0.410)	0.394
Type 2 diabetes^c,d^	−0.090	(−0.249 to 0.070)	0.241
SBP, mm Hg^b^	1.076	(0.200–1.951)	0.016
DBP, mm Hg^b^	0.667	(0.123–1.211)	0.016
TG, mg/dL^b^	0.034	(0.009–0.058)	0.008
Total cholesterol, mg/dL^b^	0.700	(−1.072 to 2.472)	0.439
HDL cholesterol, mg/dL^b^	−0.621	(−1.323 to 0.081)	0.083
LDL cholesterol, mg/dL^b^	0.004	(−0.010 to 0.018)	0.577
Uric acid, mg/dL^b^	0.080	(0.021–0.139)	0.007
eGFR, mL/min/1.73 m^2 b^	−0.166	(−0.899 to 0.566)	0.656
HbA1c, % (NGSP)^b^	−0.040	(−0.081 to −0.002)	0.060

BMI, body mass index; DBP, diastolic blood pressure; GRS, genetic risk score; eGFR, estimated glomerular filtration rate; HbA1c, hemoglobin-A1c; HDL cholesterol, high-density-lipoprotein cholesterol; J-MICC Study, Japan Multi-institutional Collaborative Cohort Study; LDL cholesterol, low-density-lipoprotein cholesterol, MR, Mendelian randomization; SBP, systolic blood pressure; TG, triglyceride.

^a^Predicted BMI was calculated using the BMI-related SNPs in Japanese, where each allele dosage was weighted by the per-allele change in 1 unit increase in BMI.

^b^The effects of BMI on quantitative cardiometabolic traits were examined by the two-stage least squares estimator method with adjustments for age and sex.

^c^The effects of BMI on incident CVDs and type 2 diabetes were examined by two-stage regressions with adjustments for age and sex.

^d^Coronary artery disease and ischemic stroke were detected by self-reported medical history.

Results in **bold** indicate significant associations with cardiometabolic traits (*P* < 0.05/12 = 0.00417).

### Two-sample MR analyses of associations of BMI with CVDs in Japanese

**Figure 2.**
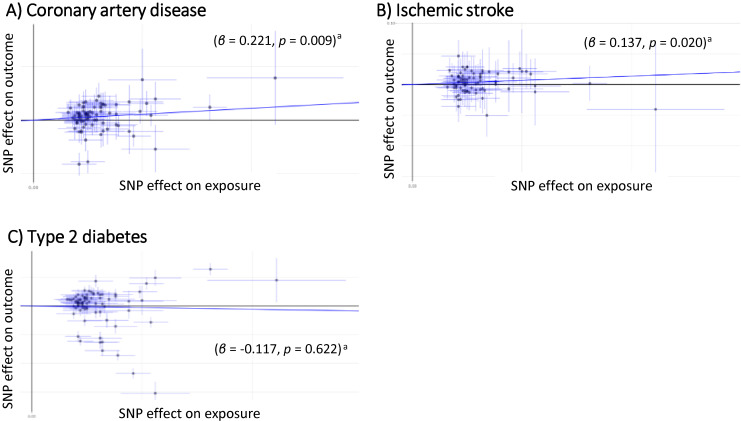
Scatter plot of the two-sample Mendelian randomization analysis showing the estimated causal associations of BMI with cardiometabolic binary traits: (**A**) coronary artery disease; (**B**) ischemic stroke; (**C**) type 2 diabetes using the IVW method. BMI, body mass index; IVW method, Inverse-Variance Weighted method. ^a^Statistical significance threshold after the Bonferroni’s adjustment was set at a two-tailed *P* value of <0.05/12 = 0.00417.

**Figure 3.**
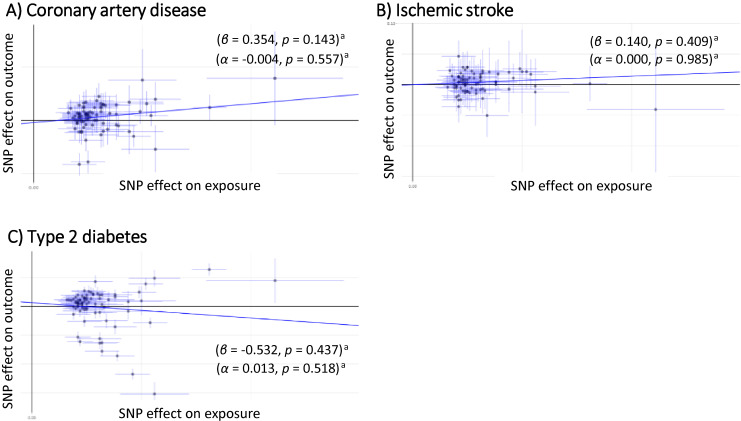
Scatter plot of the two-sample Mendelian randomization analysis showing the estimated causal associations of BMI with cardiometabolic binary traits: (**A**) coronary artery disease; (**B**) ischemic stroke; (**C**) type 2 diabetes using the MR–Egger method. BMI, body mass index; MR, Mendelian randomization. ^a^Statistical significance threshold after the Bonferroni’s adjustment was set at a two-tailed *P* value of <0.05/12 = 0.00417.

The IVW-MR analyses based on 85 SNPs as IVs for BMI demonstrated that weighted GRS of BMI was not significantly associated with risks of CAD and IS (β = 0.221; 95% CI, 0.045–0.386; *P* = 0.009 and β = 0.137; 95% CI, 0.009–0.245; *P* = 0.020, respectively) (significance level: *P* < 0.05/12 = 0.00417). The corresponding ORs were 1.247 (95% CI, 1.058–1.471) and 1.147 (95% CI, 1.021–1.287) (Figure 2). The MR–Egger analyses demonstrated the same direction of effects of the weighted GRS of BMI on those diseases, but those associations were not significant for CAD and IS (β = 0.354; 95% CI, −0.068 to 0.923; *P* = 0.143 with α = −0.004; 95% CI, −0.018 to 0.010; *P* = 0.557, and β = 0.140; 95% CI, −0.158 to 0.533; *P* = 0.409 with α = 0.000; 95% CI, −0.010 to 0.010; *P* = 0.985, respectively). The corresponding ORs were 1.425 (95% CI, 0.888–2.286) and 1.150 (95% CI, 0.825–1.603) (Figure 3).

### Two-sample MR analyses of associations of BMI with risk factors for CVDs in Japanese

**Figure 4.**
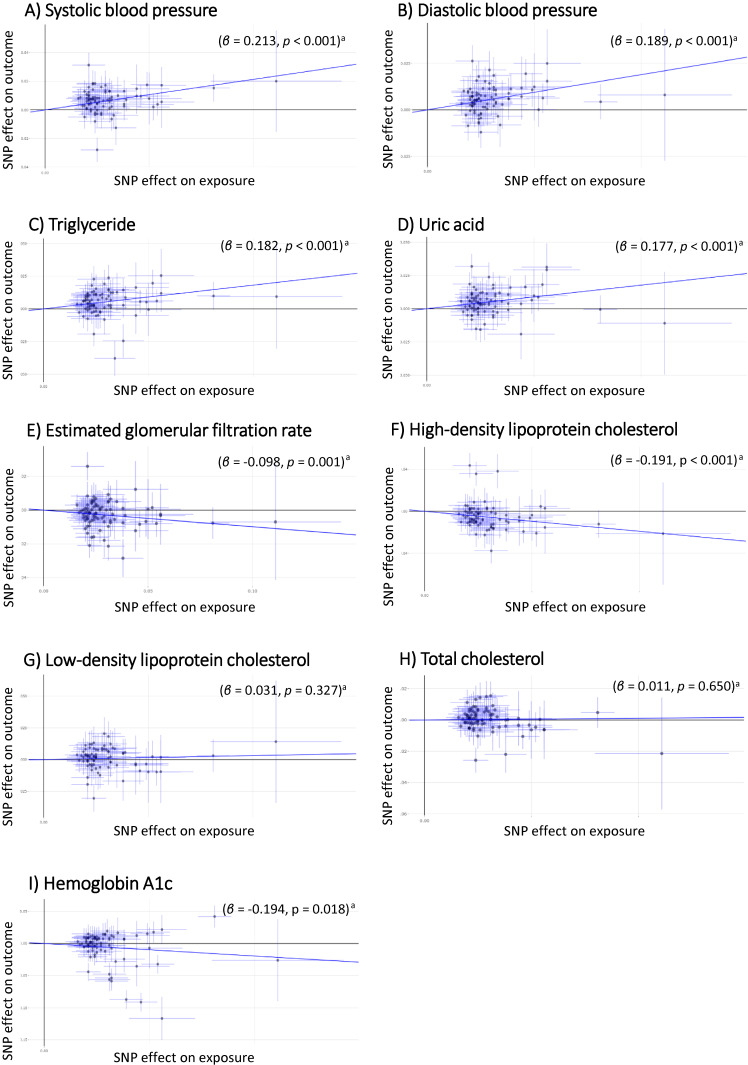
Scatter plot of the two-sample Mendelian randomization analysis showing the estimated causal associations of BMI with cardiometabolic quantitative traits: (**A**) systolic blood pressure; (**B**) diastolic blood pressure; (**C**) triglyceride; (**D**) uric acid; (**E**) estimated glomerular filtration rate; (**F**) high-density-lipoprotein cholesterol; (**G**) low-density-lipoprotein cholesterol; (**H**) total cholesterol; (**I**) hemoglobin A1c using the IVW method. BMI, body mass index; IVW method, Inverse-Variance Weighted method. ^a^Statistical significance threshold after the Bonferroni’s adjustment was set at a two-tailed *P* value of <0.05/12 = 0.00417.

**Figure 5.**
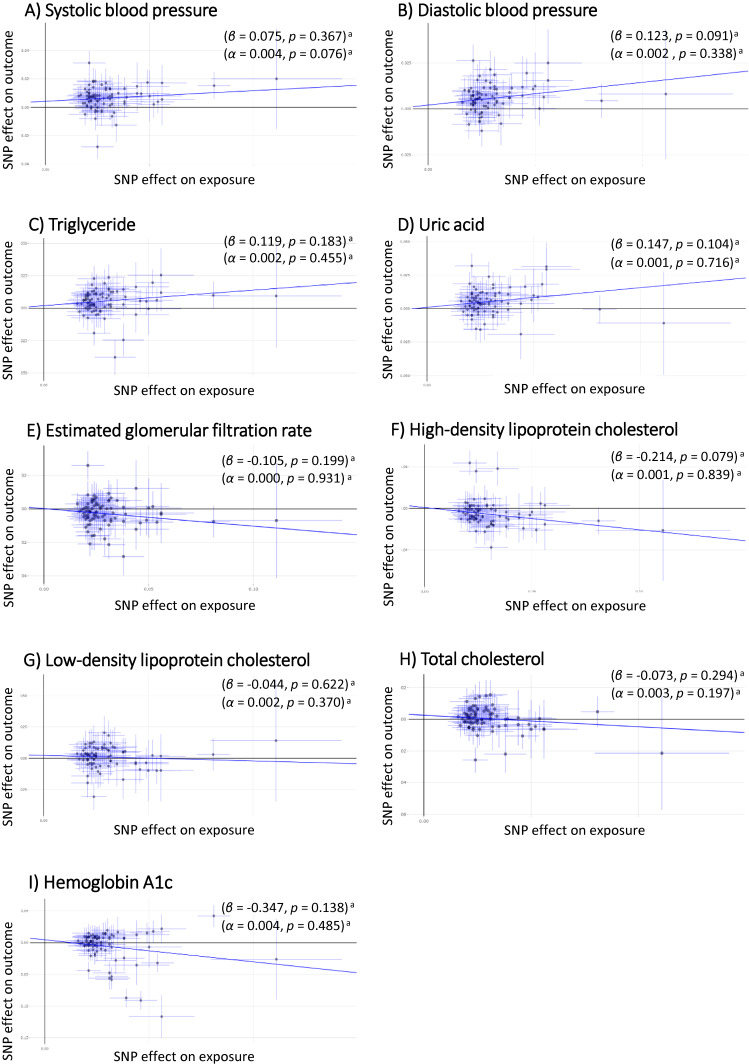
Scatter plot of the two-sample Mendelian randomization analysis showing the estimated causal associations of BMI with cardiometabolic quantitative traits: (**A**) systolic blood pressure; (**B**) diastolic blood pressure; (**C**) triglyceride; (**D**) uric acid; (**E**) estimated glomerular filtration rate; (**F**) high-density-lipoprotein cholesterol; (**G**) low-density-lipoprotein cholesterol; (**H**) total cholesterol; (**I**) hemoglobin A1c using the MR–Egger method. BMI, body mass index; MR, Mendelian randomization. ^a^Statistical significance threshold after the Bonferroni’s adjustment was set at a two-tailed *P* value of <0.05/12 = 0.00417.

**Figure 6.**
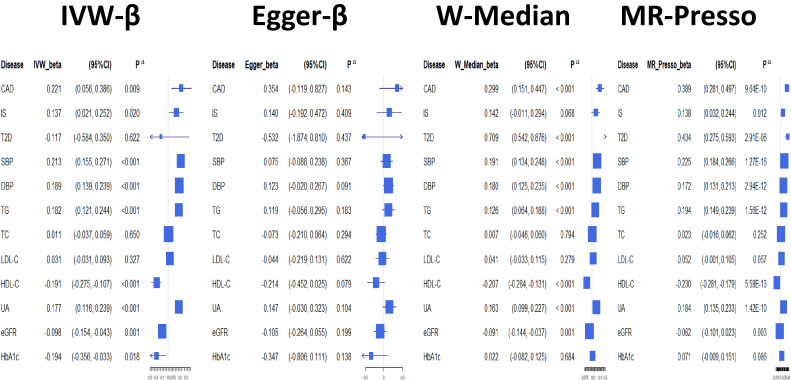
Forest plots for the two-sample Mendelian randomization analysis showing the estimated causal associations of BMI with cardiometabolic traits. BMI, body mass index; CAD, coronary artery disease; DBP, diastolic blood pressure; eGFR, estimated glomerular filtration rate; Egger-β, Mendelian randomization-Egger; HbA1c, hemoglobin A1c; HDL, high-density-lipoprotein cholesterol; IS, ischemic stroke; IVW-β, Inverse-Variance Weighted Mendelian randomization; LDL, low-density-lipoprotein cholesterol; MR-PRESSO, Mendelian Randomization Pleiotropy RESidual Sum and Outlier; SBP, systolic blood pressure; T2D, type 2 diabetes; TC, total cholesterol; TG, triglyceride; UA, uric acid; W-Median, weighted median Mendelian randomization. ^a^Statistical significance threshold after the Bonferroni’s adjustment was set at a two-tailed *P* value of <0.05/12 = 0.00417.

The IVW-MR analyses based on the same 85 SNPs for BMI demonstrated that higher BMI was significantly associated with higher SBP, DBP, TG, and UA (β = 0.213; 95% CI, 0.030–0.155, β = 0.189; 95% CI 0.139–0.239, β = 0.182; 95% CI, 0.033–0.118, and β = 0.177; 95% CI, 0.113–0.239, respectively, all with *P* < 0.001 [significance level: *P* < 0.05/12 = 0.00417]). Higher BMI was also associated with lower levels of HDL cholesterol (β = −0.191; 95% CI, −0.275 to −0.107, *P* < 0.001) and lower levels of eGFR (β = −0.098; 95% CI, −0.146 to −0.036, *P* = 0.001) (Figure 4). The MR–Egger analyses demonstrated no associations at most, while the directions of effects were well retained (Figure 5). No regression intercept (α) significantly differed from zero (significance level: *P* < 0.05/12 = 0.00417) (Figure 5). Meanwhile, both methods of the MR analyses revealed that BMI was not associated with type 2 diabetes, TC, LDL cholesterol, or HbA1c. The forest plot for the overall summary of the MR analyses is provided in Figure 6.

### Sensitivity analyses

We conducted sensitivity analyses using the weighted median MR and MR-PRESSO to assess the robustness of the IVW-MR results and to rule out the possibility of false-positives. The both results from the weighted median MR and MR-PRESSO were similar to those observed in IVW-MR other than type 2 diabetes and HbA1c. In contrast to the results from IVW-MR, BMI was positively associated with type 2 diabetes in the weighted median MR and MR-PRESSO (Figure 6 and eTable 4). The results from MR-PRESSO showed that number of the possible outliers in IVs was relatively large in type 2 diabetes (*N* = 37) and HbA1c (*N* = 12) (eTable 4). In the MVMR analyses, DBP was significantly associated with CAD (eTable 5). When we excluded SNPs which had strong associations with both BMI and other cardiometabolic traits in GWAS of Asians, the results from IVW-MR and Egger-MR did not largely change. However, associations of BMI with type 2 diabetes and HbA1c in Egger-MR were stronger than those calculated by original 85 SNPs; higher BMI was nominally significantly inversely associated with type 2 diabetes and HbA1c (β = −1.911; 95% CI, −3.482 to −0.340, *P* = 0.017 and β = −0.794; 95% CI, −1.289 to −0.299, *P* = 0.002, respectively). Then, the regression intercept (α) of type 2 diabetes, HbA1c, and SBP were significantly differed from zero (α = 0.050, *P* = 0.020; α = 0.017, *P* = 0.011; and α = 0.006, *P* = 0.021, respectively).

## DISCUSSION

The present study revealed that the GRS of BMI was not significantly associated with any cardiometabolic traits by an individual-level MR approach among a population of 14,083 Japanese. Using a two-sample MR with IVW approach among 173,430 Japanese, we showed that higher BMI was associated with higher risks of a variety of cardiometabolic traits (higher SBP, DBP, TG, and UA, and lower HDL cholesterol and eGFR). In this population, the association of BMI with type 2 diabetes was inconsistent among the MR analysis method, including the directions. BMI was not significantly associated with type 2 diabetes in the individual-level MR approach and in two-sample MR with IVW and Egger methods. Whereas BMI was positively associated with type 2 diabetes in two-sample MR with the weighted median MR and MR-PRESSO, the direction of the association was opposite to those of the two-sample MR with IVW and Egger-MR. To the best of our knowledge, the present study is the first to investigate the association of BMI with cardiometabolic traits using the MR approach with a large Japanese sample. Our results add evidence suggesting that, even among the Japanese, an Asian population with a low level of obesity, higher BMI could be causally associated with the development of a variety of known CVD risk factors, in line with the results of European studies.^40^

The observed positive associations of obesity or higher BMI with higher blood pressure and TG, and inverse associations with HDL cholesterol and eGFR are biologically plausible. Obesity or higher BMI-related inflammation, visceral fat accumulation, oxidative stress,^9^ circulation of saturated fatty acids, insulin resistance,^10^ and endothelial dysfunction^11^ contribute to these conditions. Although those previously reported associations could have been influenced by reverse/bidirectional causality, and/or unadjusted confounders in observational studies, our results in two-sample MR support the possibility that they are causal. The reason for the no association in the present individual-level MR may be for the relatively small sample size of the J-MICC study. As most of the directions of the associations were similar between individual-level MR and two-sample MR, further studies with larger sample sizes may clarify those associations.

Meanwhile, the major difference in our findings, including the direction of the associations, compared with findings from Europeans, was that there was inconsistent association between BMI and type 2 diabetes depending on the MR analysis method, and no association of BMI with HbA1c in our population. Previous MR studies using large European datasets revealed the strong causal effects of overall and abdominal obesity on both the development of type 2 diabetes and glycemic traits (fasting glucose, fasting insulin, and HbA1c).^40^^,^^41^ Although the effect of obesity on HbA1c was relatively small in one study,^41^ both results supported the assertion that obesity causes type 2 diabetes among Europeans through the deterioration of glycemic control and insulin resistance. Among the Japanese, consistent relationships were found between obesity or higher BMI and incident type 2 diabetes in epidemiological studies,^4^^–^^6^ but the relationships could be complicated.^29^ In a recent large Japanese GWAS, Akiyama et al compared effect sizes of BMI-associated variants on susceptibility to type 2 diabetes.^29^ Of the 193 variants, they detected genome-wide significant levels (*P* < 5.0 × 10^−8^) of association for type 2 diabetes, and found that only 5 of 20 variants had a positive association, whereas 15 variants had a negative one.^29^ Those variants negatively associated with type 2 diabetes were reported to influence insulin processing and secretion without a detectable change in fasting glucose levels among non-diabetic participants who were mainly recruited in European countries.^42^ For example, *CDKAL1* and *HHEX* had only large negative effects on insulinogenic index, but with very modest effects on fasting glucose levels.^42^ In the study by Akiyama et al, they found a strong genetic correlation between BMI and type 2 diabetes, as well as ischemic cardiovascular diseases, in the data from BBJ GWAS and 33 other GWAS of Asians using bivariate linkage disequilibrium (LD) score regression.^43^ However, our results could not replicate the correlation between higher BMI and susceptibility to type 2 diabetes among the Japanese. The SNPs which had associations both with BMI and other cardiometabolic traits in GWAS of Asians (12 out of total 85 SNPs), and the relatively large number of outlier (37 out of total 85 SNPs) detected by MR-PRESSO could have important role to understand the association. On the other hand, the shared 12 SNPs may be considered as common genetic factors between the two traits (eg, FTO), and their exclusion may potentially lead to bias. The result after excluding those SNPs showed significant inverse associations of BMI with type 2 diabetes and HbA1c, but the horizontal pleiotropy of the IVs was observed in both associations. Those causality may not be reliable.

The results of the present study could also be explained by the different characteristics of diabetes in this ethnic group from those in Europeans. The fact that we could not find a significant relationship between BMI and type 2 diabetes among the Japanese in individual-level MR and two-sample MR analyses by IVW-β and Egger-MR. The onset of type 2 diabetes in the Japanese and other East Asians is characterized primarily by less obesity and earlier β-cell dysfunction than that in Europeans. Given the limited β-cell capacity in East Asians, they are susceptible to even a small decline of insulin sensitivity by a small weight gain. Meanwhile, type 2 diabetes among Europeans is mainly caused by decreased insulin sensitivity, which is more closely related to obesity. In addition to that, the higher proportion of elderly (≥65 years) among Japanese diabetics could be another reason for the different association with higher BMI from those in Europeans. The International Diabetes Federation reported that, in 2019, Japan had the sixth largest number of diabetics aged 65 years or older globally.^44^ In addition, a recent MR study by Noordam et al reported that the association between BMI and type 2 diabetes was attenuated with increasing age of receiving a diagnosis of type 2 diabetes.^45^ Although it is difficult to directly compare the age at which diabetes was diagnosed, the mean age at baseline of the current study participants from BBJ is higher than that of the UK Biobank (63 years^29^ vs 57 years^46^). As Wang et al pointed out the possible difference of overall and abdominal obesity in terms of the mechanisms by which type 2 diabetes develops among Chinese Han individuals,^47^ the distribution of adiposity could have influenced the results. Differences in the first-choice diabetes drugs could also contribute to the difference of the association between BMI and diabetes in Japanese and those in Europeans. For example, sulfonylurea, the first-choice drug for diabetes in the United Kingdom,^48^ is associated with weight gain, but dipeptidyl peptidase-4 inhibitors, which are widely used in Japan,^49^ are not.

In the two-sample MR analysis in this study, the results from the IVW method showed more prominent associations than those from the MR–Egger analysis. In previous reports, the IVW method is recommended for two-sample MR analysis when sample sizes are large.^50^ As sample sizes in the current study were sufficiently large, we think that the results from the IVW method may be reliable estimates for causal associations between BMI and disease outcomes. As the precision of MR–Egger estimates is considered to depend on the variability of the genetic associations of IVs with risk factors,^51^ the weaker associations in MR–Egger analyses suggest large variability in this Japanese sample. Meanwhile, MR–Egger regression is a novel method of assessing the horizontal pleiotropy of IVs. As no regression intercept (α) significantly differed from zero (significance level: *P* < 0.05/12 = 0.00417) in the current study, we considered that there may not be substantial horizontal pleiotropy in the results. Although no association between weighted GRS for BMI and potential confounders (smoking, drinking or daily physical activities) suggests that there is little possibility of the violation of MR assumption by IV-confounder associations, MVMR analyses suggest that BMI related SNPs could be associated with CAD through other cardiometabolic pathways. The results of sensitivity analyses by weighted median MR and MR-PRESSO support the possibility that most of the cardiometabolic traits with significant associations observed in the IVW-MR have causal relationships. However, as possible bias in the association of BMI with type 2 diabetes was suggested, the direct effects of BMI-associated SNPs on cardiometabolic traits should be clarified in detail in further investigations. Considering the rather clustered distribution of SNP–exposure as well as SNP–outcome associations in the present two-sample MR analyses, further investigations of these associations in near future are warranted.

The findings of the current study should be interpreted with caution. First, the difference in circumstances for BMI measurement in the J-MICC Study and BBJ could have influenced the results. Because most of the participants in the J-MICC Study were recruited in the community, whereas those of BBJ were measured in clinical settings, BMI levels could have been influenced by the target disease and/or the medical treatment in the latter situation. However, our results from individual-level MR analyses and two-sample MR analyses were similar in the direction, suggesting that higher BMI may be causally associated with cardiometabolic traits other than diabetes and HbA1c in the Japanese. As no association in the individual-level MR could be related to the relatively small sample size, further studies with larger sample sizes may declare the associations. Second, CVDs were detected based on only self-reported medical history in the J-MICC Study. The lack of associations between BMI and CVDs in the individual-MR analysis of this study could have been influenced by the difference of CVDs measurement. As the result by multivariable MR showed that another risk factor was associated with CHD, further studies should be performed to confirm the causal associations. Third, we used only BMI to detect tendency to be obese, but different distributions of adiposity could have influenced the results. Fourth, we should be careful about the “winner’s curse,”^52^ because we used the SNPs discovered from GWAS meta-analysis mainly consisted of BBJ study as IVs. As a result of false discovery associated with this phenomenon, the magnitude and direction of bias should be observed away from the null. Fifth, as there were considerably large overlaps in samples analyzed for the individual MR and two-sample MR in our study, the assumption of independence between the gene-exposure and gene-outcome association could be violated in the presence of confounding. However, based on the simulations by Minelli et al,^50^ in which they assessed the performance of MR analysis by mimicking a typical study data in the United Kingdom Biobank, two-sample MR methods can be safely used for such overlapped data from large biobanks. That being said, the interpretation of results from MR-Egger analysis requires caution because they are biased, reflecting the direction and magnitude of the confounding.^50^ In another aspect, with overlapped data, the MR estimates would not necessarily be biased toward the null, given the upwardly biased betas from the discovery GWAS (called as “winner’s curse”).^53^ Despite these limitations, the J-MICC Study has a wealth of information on genotypes related to BMI and a variety of cardiometabolic traits. This allowed us to examine relationships between BMI and cardiometabolic traits in individual-level MR analyses. The huge sample size with plenty of information from BBJ, the JPHC study, and TMM enabled us to confirm the causal associations using the two-sample MR method in the Japanese.

In summary, the present study is the first to comprehensively examine the association between BMI and cardiometabolic outcomes in the Japanese, which may provide potentially useful information for disease prevention in the Japanese. Further investigations with a larger or independent population are now warranted.

## SUPPLEMENTARY MATERIAL

eTable 1. SNPs used in calculating a weighted genetic risk score for BMI in the J-MICC Study participants

eTable 2. The phenotypical association between BMI and cardiometabolic traits in the J-MICC Study

eTable 3. The associations of weighted genetic risk score for BMI with potential confounders in the J-MICC study

eTable 4. Associations of the predicted BMI and cardiometabolic traits by two-sample MR using MR-PRESSO analyses

eTable 5. Associations of the predicted BMI and cardiometabolic traits by 2 sample MR using the multivariable MR analyses
