# Supplementary material for: BMI and Cardiometabolic Traits in Japanese: A Mendelian Randomization Study
Source: J Epidemiol. 2024 Feb 5;34(2):51–62. doi: 10.2188/jea.JE20220154 (PMC10751192; doi:10.2188/jea.JE20220154)
Supplement: Supplementary file 1 [file je-34-051-s001.pdf]

eTable 1. SNPs used in calculating a weighted genetic risk score for BMI in the J-MICC Study participants

Positional candidate genes	SNP ID	proxy SNP ID	Chr	Position (bp)	Alleles Ref/Alt	Ref_frq	Alt_frq	BMI		
								beta	SE	*P*
FGR,IFI6	rs2076463		1	27971092	A/G	0.721	0.279	0.024	0.044	0.579
GON4L	rs860295		1	155767708	A/G	0.752	0.248	0.144	0.045	0.001
LOC101928778,SEC16B	rs633715		1	177852580	T/C	0.773	0.227	0.133	0.047	0.005
BRINP3	rs491055		1	190308834	G/A	0.352	0.648	-0.012	0.041	0.771
FAM150B,TMEM18	rs939584		2	621558	T/C	0.103	0.897	-0.199	0.064	0.002
SDC1,PUM2	rs111612372		2	20433218	T/C	0.802	0.198	0.121	0.049	0.013
ADCY3,DNAJC27	rs713586		2	25158008	C/T	0.48	0.52	-0.122	0.039	0.002
PRKD3,QPCT	rs6734118		2	37559355	A/C	0.46	0.54	-0.052	0.039	0.185
LOC101929596,HNRNPLL	rs77489951		2	38750287	C/T	0.94	0.06	0.047	0.081	0.560
NRXN1	rs10174398		2	51195601	C/T	0.415	0.585	0.104	0.040	0.009
PSME4	rs10208649		2	54161363	T/C	0.987	0.013	-0.394	0.171	0.021
LINC01122	rs10197655		2	58791420	A/G	0.406	0.594	0.137	0.039	<0.001
LRP1B	rs12617004		2	142615136	G/C	0.613	0.387	-0.020	0.040	0.620
STK39	rs2390669		2	169091942	A/C	0.768	0.232	-0.077	0.046	0.095
CWC22,SCHLAP1	rs6433857		2	181517996	C/T	0.676	0.324	-0.054	0.041	0.195
VGLL4	rs2574704		3	11655381	C/T	0.349	0.651	-0.045	0.041	0.271
CCK	rs8192473		3	42299399	C/T	0.904	0.096	-0.045	0.066	0.494
NEK4	rs11130319		3	52755592	T/A	0.475	0.525	-0.063	0.039	0.107
IGF2BP2	rs4686392		3	185524081	A/G	0.671	0.329	-0.072	0.041	0.082
GNPDA2,GABRG1	rs1996023		4	45164637	G/T	0.278	0.722	0.091	0.043	0.035
RGS7BP,FAM159B	rs1035491		5	63962177	A/G	0.731	0.269	-0.032	0.044	0.463

POC5	rs6881648		5	74991849	C/A	0.43	0.57	-0.025	0.039	0.520
LINC00461	rs1846974		5	87969927	G/A	0.532	0.468	0.008	0.039	0.828
PCSK1	rs10062657		5	95867908	A/C	0.392	0.608	0.111	0.040	0.006
ZNF608,LOC101927421	rs4357030		5	124316031	T/C	0.455	0.545	-0.072	0.039	0.067
JADE2	rs329120		5	133861756	C/T	0.622	0.378	0.056	0.040	0.159
CDKAL1	rs35261542		6	20675792	C/A	0.586	0.414	-0.066	0.040	0.096
HLA-DRA,HLA-DRB5	rs183975233		6	32437160	T/A	0.616	0.384	0.004	0.040	0.926
GRM4,HMGA1	rs6913361		6	34179390	G/A	0.131	0.869	-0.012	0.058	0.834
TFAP2B	rs2206271		6	50786008	T/A	0.648	0.352	0.090	0.041	0.027
EYS	rs148546399		6	64705610	G/A	0.964	0.036	0.277	0.105	0.008
RGS17	rs9397585	rs6927884	6	153397429	A/T	0.654	0.356	-0.014	0.041	0.725
AUTS2	rs6947395	rs10266126	7	69375390	G/T	0.807	0.193	-0.042	0.049	0.391
LINC01392,TFEC	rs143665886	rs7776991	7	115379197	A/T	0.571	0.429	-0.036	0.039	0.359
LOC102724612,LINC01289	rs77636220	rs16930598	8	64604836	G/A	0.789	0.211	0.079	0.048	0.098
HNF4G,LINC01111	rs28857569	rs80276256	8	76632750	A/C	0.689	0.311	0.089	0.042	0.035
KIAA1429	rs4366055	rs10111287	8	95578426	C/T	0.51	0.49	-0.132	0.039	0.001
CDKN2B-AS1,DMRTA1	rs7020996		9	22129579	C/T	0.558	0.442	0.042	0.039	0.285
SLC28A3,NTRK2	rs10868215		9	87234111	T/C	0.676	0.324	0.050	0.041	0.230
ZNF169,NUTM2F	rs3932549		9	97073588	C/A	0.305	0.695	-0.046	0.042	0.280
GAPVD1,MAPKAP1	rs5015933		9	128137418	C/T	0.463	0.537	0.108	0.039	0.005
CDC123,CAMK1D	rs10795945		10	12302607	C/T	0.454	0.546	0.113	0.039	0.004
CACNB2	rs7912454		10	18584792	A/G	0.827	0.173	0.0003	0.052	0.994
HERC4	rs80117551		10	69834828	C/T	0.742	0.258	-0.182	0.044	<0.001
HHEX,EXOC6	rs1832886		10	94477539	A/G	0.198	0.802	-0.105	0.049	0.032
FRAT2,RRP12	rs12569457		10	99096676	C/T	0.805	0.195	0.097	0.049	0.049

HIF1AN,PAX2	rs2495707		10	102425949	A/G	0.536	0.464	-0.017	0.039	0.663
C10orf32-ASMT	rs4409766		10	104616663	T/C	0.705	0.295	0.195	0.043	<0.001
TCF7L2	rs7903146		10	114758349	C/T	0.96	0.04	0.060	0.100	0.546
MIR5694,FGFR2	rs1907240		10	122897959	A/G	0.308	0.692	0.031	0.042	0.463
BUB3,GPR26	rs1568079		10	125251751	T/A	0.643	0.357	-0.046	0.041	0.260
KCNQ1	rs60808706		11	2857233	G/A	0.605	0.395	0.092	0.040	0.020
LMO1,STK33	rs16937956		11	8404501	G/A	0.43	0.57	0.056	0.040	0.158
BDNF	rs11030100		11	27677586	G/T	0.596	0.404	-0.265	0.040	<0.001
FNBP4	rs11602339		11	47761471	C/T	0.676	0.324	0.068	0.041	0.103
FAM60A	rs80234489		12	31441179	A/C	0.804	0.196	-0.056	0.049	0.255
FAIM2	rs3205718		12	50261809	C/T	0.716	0.284	0.154	0.043	<0.001
MAP3K12	rs77511173		12	53883537	T/C	0.895	0.105	0.026	0.063	0.675
ALDH2,MAPKAPK5-AS1	rs7305242		12	112256762	C/T	0.365	0.635	0.258	0.040	<0.001
LINC01065,LINC00558	rs9568867		13	54107352	G/A	0.778	0.222	-0.020	0.047	0.666
NID2	rs75766425	rs79823890	14	52511969	G/T	0.872	0.128	0.165	0.059	0.005
UNC79	rs729050	rs12432051	14	94075104	C/T	0.59	0.41	0.027	0.04	0.498
TCF12	rs2593235		15	57541201	G/A	0.608	0.392	-0.035	0.040	0.382
VPS13C	rs72749754		15	62319432	G/C	0.77	0.23	-0.065	0.046	0.160
ADCY9	rs2540034		16	4022694	C/T	0.68	0.32	0.086	0.042	0.040
GPR139,GP2	rs12597682		16	20258432	C/A	0.818	0.182	-0.177	0.050	<0.001
IL27,NUPR1	rs62034325		16	28538640	A/G	0.88	0.12	0.167	0.060	0.005
FTO	rs11642015		16	53802494	C/T	0.804	0.196	0.444	0.049	<0.001
ZFHX3	rs4788694		16	73070083	G/C	0.319	0.681	0.159	0.042	<0.001
BPTF	rs4790981		17	65921834	G/A	0.298	0.702	-0.042	0.042	0.327
RIT2,SYT4	rs1518170		18	40708905	C/T	0.311	0.689	-0.045	0.042	0.289

PMAIP1,MC4R	rs6567160	18	57829135	T/C	0.795	0.205	0.145	0.048	0.003
GIPR	rs35560038	19	46175046	T/A	0.384	0.616	0.123	0.040	0.002
ZNF133	rs16978956	20	18288165	A/G	0.808	0.192	0.125	0.050	0.012
LINC01441,CBLN4	rs2247627	20	54145086	G/A	0.557	0.443	-0.016	0.039	0.681
TAF4	rs6089584	20	60564086	C/G	0.344	0.656	0.076	0.041	0.062
LOC400867,LOC101928435	rs9983113	21	40315316	T/G	0.236	0.764	0.025	0.045	0.578
TNRC6B	rs139913	22	40713861	T/A	0.535	0.465	-0.052	0.039	0.185
DMD	rs1379871	X	31854782	C/G	0.23	0.77	-0.128	0.049	0.009
HSD17B10,HUWE1	rs6529684	X	53542107	A/G	0.624	0.376	-0.009	0.044	0.841
IL13RA1	rs3121672	X	117916370	T/C	0.677	0.323	0.011	0.045	0.806
GPR101	rs1190736	X	136113464	C/A	0.78	0.22	-0.215	0.049	<0.001
FAM58A,DUSP9	rs5945324	X	152894551	G/C	0.76	0.24	-0.048	0.048	0.310

Alt, alternative allele; BMI, body mass index; bp, base pairs; Chr, chromosome; frq, frequency; ID, identification; J-MICC Study, Japan Multi-Institutional Collaborative Cohort Study; Ref, reference allele; SE, standard error; SNP, single-nucleotide polymorphism.

eTable 2. The phenotypical association between BMI and cardiometabolic traits in the J-MICC Study

Trait	Odds ratio	95% confidence interval	*P*
Cardiometabolic binary traits			
Coronary artery disease, history	**1.065**	**(1.034–1.097)**	**<0.001**
Ischemic stroke, history	**1.080**	**(1.042–1.119)**	**<0.001**
T2DM	**1.153**	**(1.133–1.173)**	**<0.001**
	beta	95% confidence interval	*P*
Cardiometabolic quantitative traits			
SBP, mm Hg	**1.503**	**(1.403–1.603)**	**<0.001**
DBP, mm Hg	**0.913**	**(0.851–0.976)**	**<0.001**
TG, mg/dL	**6.443**	**(5.942–6.944)**	**<0.001**
Total cholesterol, mg/dL	**1.080**	**(0.883–1.277)**	**<0.001**
HDL cholesterol, mg/dL	**-1.452**	**(-1.532 to -1.371)**	**<0.001**
LDL cholesterol, mg/dL	**1.377**	**(1.196–1.557)**	**<0.001**
Uric acid, mg/dL	**0.082**	**(0.075–0.088)**	**<0.001**
eGFR, mL/min/1.73 m^2	-0.101	(-0.184 to -0.018)	0.017
HbA1c, % (NGSP)	**0.039**	**(0.034–0.043)**	**<0.001**

BMI, body mass index; DBP, diastolic blood pressure; eGFR, etimated glomerular filtration rate; HbA1c, Hemoglobin-A1c; HDL cholesterol, high-density lipoprotein cholesterol; J-MICC Study, Japan Multi-institutional Collaborative Cohort Study; LDL cholesterol, low-density lipoprotein cholesterol; SBP, systolic blood pressure; TG, triglycerides; T2DM, type 2 diabetes mellitus.
Results in **bold** indicate significant associations with cardiometabolic traits (*P*<0.05/12 = 0.00417).

eTable 3. The associations of weighted genetic risk score for BMI with potential confounders in the J-MICC study

	beta	SE	P^a
Smoking, BI	-21.23	29.74	0.475
Alcohol, g/day	1.49	2.38	0.531
PA, METs/day	1.54	0.94	0.101

BI, Brinkman Index, BMI, body mass index; J-MICC Study, Japan Multi-institutional Collaborative Cohort Study; MET, metabolic equivalent of task; PA, physical activity; SE, standard error.

[a]Statistical significance threshold was set at a two-tailed *P* value of <0.05.

eTable 4. Associations of the predicted BMI and cardiometabolic traits by two-sample MR using MR-PRESSO analyses

Trait	number of outliers	Global-P[a]	*β*[b]	SE	*P*[c]
Coronary artery disease	10	**0.018**	**0.389**	**0.055**	**9.64E-10**
Ischemic stroke	2	1.842	0.138	0.054	0.012
Type 2 diabetes	37	**0.012**	**0.434**	**0.081**	**2.91E-06**
SBP, mm Hg	7	**0.024**	**0.225**	**0.021**	**1.27E-16**
DBP, mm Hg	11	0.258	**0.172**	**0.021**	**2.94E-12**
TG, mg/dL	6	0.066	**0.194**	**0.023**	**1.56E-12**
Total cholesterol, mg/dL	3	0.126	0.023	0.020	0.252
HDL cholesterol, mg/dL	6	1.254	**-0.230**	**0.026**	**5.58E-13**
LDL cholesterol, mg/dL	2	0.186	0.052	0.027	0.057
Uric acid, mg/dL	10	**0.024**	**0.184**	**0.025**	**1.42E-10**
eGFR, mL/min/1.73 m^2	8	0.090	**-0.062**	**0.02**	**0.003**
HbA1c, % (NGSP)	12	**0.012**	0.071	0.041	0.086

BMI, body mass index; DBP, diastolic blood pressure; GRS, genetic risk score; eGFR, estimated glomerular filtration rate; HbA1c, hemoglobin-A1c; HDL cholesterol, high-density-lipoprotein cholesterol; J-MICC Study, Japan Multi-institutional Collaborative Cohort Study; LDL cholesterol, low-density-lipoprotein cholesterol; MR, Mendelian randomization; MR-PRESSO, Mendelian Randomization Pleiotropy RESidual Sum and Outlier; NGSP, National Glycohemoglobin Standardization Program; SBP, systolic blood pressure; SE, standard error; TG, triglyceride.

[a]The overall horizontal pleiotropy was tested with the MR-PRESSO global test.

[b]Outlier-corrected exposure β values after excluding outliers based on the outlier test until the MR-PRESSO global test indicated no significant influence of outliers (Global-P $\geq 1.0\times10^{-6}$)

[c]Outlier-corrected P value after excluding outliers based on the outlier test until the MR-PRESSO global test indicated no significant influence of outliers (Global-P $\geq 1.0\times10^{-6}$)

Results in **bold** indicate significant associations with cardiometabolic traits ($P < 0.05/12 = 0.00417$).

eTable 5. Associations of the predicted BMI and cardiometabolic traits by 2 sample MR using the multivariable MR analyses

	Coronary artery disease				Ischemic stroke			
Trait	beta	SE	t	*P*	beta	SE	t	*P*
BMI	0.336	0.260	1.292	0.200	0.195	0.198	0.987	0.327
SBP, mm Hg	2.871	5.624	0.510	0.611	0.485	4.264	0.114	0.910
DBP, mm Hg	**1.373**	**0.453**	**3.034**	**0.003**	0.338	0.344	0.983	0.329
TG, mg/dL	1994.350	2457.750	0.812	0.420	-27.362	1888.500	-0.015	0.988
HDL cholesterol, mg/dL	0.702	0.267	2.631	0.010	0.430	0.397	1.085	0.281
LDL cholesterol, mg/dL	-187.040	210.240	-0.890	0.377	-52.888	1788.400	-0.030	0.977
Uric acid, mg/dL	-0.897	0.521	-1.723	0.089	0.023	0.203	0.145	0.909
eGFR, mL/min/1.73 m^2	-1787.570	2322.470	-0.770	0.444	69.554	159.105	0.437	0.663
HbA1c, % (NGSP)	-0.604	0.291	-2.077	0.041	-0.037	0.222	-0.168	0.867

DBP, diastolic blood pressure; eGFR, estimated glomerular filtration rate; HbA1c, hemoglobin-A1c; HDL cholesterol, high-density-lipoprotein cholesterol; J-MICC Study, Japan Multi-institutional Collaborative Cohort Study; LDL cholesterol, low-density-lipoprotein cholesterol; NGSP, National Glycohemoglobin Standardization Program; SBP, systolic blood pressure; SE, standard error; TG, triglyceride.

Results in **bold** indicate significant associations with cardiometabolic traits (P <0.05/9 = 0.00556).
